# The burden, prevention and care of infants and children with congenital anomalies in sub-Saharan Africa: A scoping review

**DOI:** 10.1371/journal.pgph.0001850

**Published:** 2023-06-28

**Authors:** Aminkeng Zawuo Leke, Helen Malherbe, Emma Kalk, Ushma Mehta, Phylis Kisa, Lorenzo D. Botto, Idowu Ayede, Lee Fairlie, Nkwati Michel Maboh, Ieda Orioli, Rebecca Zash, Ronald Kusolo, Daniel Mumpe-Mwanja, Robert Serujogi, Bodo Bongomin, Caroline Osoro, Clarisse Dah, Olive Sentumbwe–Mugisha, Hamisi Kimaro Shabani, Philippa Musoke, Helen Dolk, Linda Barlow-Mosha

**Affiliations:** 1 Institute for Nursing and Health Research, Centre for Maternal, Fetal and Infant Research, Ulster University, Newtownabbey, United Kingdom; 2 Centre for Infant and Maternal Health Research, Health Research Foundation, Buea, Cameroon; 3 Research & Epidemiology, Rare Diseases South Africa NPC, Bryanston, Sandton, South Africa; 4 Centre for Infectious Disease Epidemiology & Research, School of Public Health, University of Cape Town, Cape Town, South Africa; 5 Makerere University College of Health Sciences, Kampala, Uganda; 6 Division of Medical Genetics, University of Utah, Salt Lake City, Utah, United States of America; 7 International Center on Birth Defects, University of Utah, Salt Lake City, Utah, United States of America; 8 Department of Paediatrics, College of Medicine, University of Ibadan and University College Hospital, Ibadan, Nigeria; 9 Faculty of Health Sciences, Wits Reproductive Health and HIV Institute, University of the Witwatersrand, Johannesburg, South Africa; 10 Genetics Department, Federal University of Rio de Janeiro, Rio de Janeiro, Brazil; 11 ReLAMC: Latin American Network for Congenital Malformation Surveillance, Federal University of Rio de Janeiro, Rio de Janeiro, Brazil; 12 The Botswana Harvard AIDS Institute Partnership, Gaborone, Botswana and Beth Israel Deaconess Medical Center, Boston, Massachusetts, United States of America; 13 Makerere University-Johns Hopkins University Research Collaboration, Kampala, Uganda; 14 Gulu University Faculty of Medicine: Gulu, Gulu, UG/ World Health Organization, Kampala, Uganda; 15 Kenya Medical Research Institute, Centre for Global Health Research, Nairobi, Kenya; 16 Centre de Recherche en Santé de Nouna, Nouna, Burkina Faso; 17 World Health Organization, Kampala, Uganda; 18 Neurosurgery Department, Muhimbili Orthopaedic Institute, Dar es salaam, Tanzania; PLOS: Public Library of Science, UNITED STATES

## Abstract

The aim of this scoping review was to determine the scope, objectives and methodology of contemporary published research on congenital anomalies (CAs) in sub-Saharan Africa (SSA), to inform activities of the newly established sub-Saharan African Congenital Anomaly Network (sSCAN). MEDLINE was searched for CA-related articles published between January 2016 and June 2021. Articles were classified into four main areas (public health burden, surveillance, prevention, care) and their objectives and methodologies summarized. Of the 532 articles identified, 255 were included. The articles originated from 22 of the 49 SSA countries, with four countries contributing 60% of the articles: Nigeria (22.0%), Ethiopia (14.1%), Uganda (11.7%) and South Africa (11.7%). Only 5.5% of studies involved multiple countries within the region. Most articles included CA as their primary focus (85%), investigated a single CA (88%), focused on CA burden (56.9%) and care (54.1%), with less coverage of surveillance (3.5%) and prevention (13.3%). The most common study designs were case studies/case series (26.6%), followed by cross-sectional surveys (17.6%), retrospective record reviews (17.3%), and cohort studies (17.2%). Studies were mainly derived from single hospitals (60.4%), with only 9% being population-based studies. Most data were obtained from retrospective review of clinical records (56.1%) or via caregiver interviews (34.9%). Few papers included stillbirths (7.5%), prenatally diagnosed CAs (3.5%) or terminations of pregnancy for CA (2.4%).This first-of-a-kind-scoping review on CA in SSA demonstrated an increasing level of awareness and recognition among researchers in SSA of the contribution of CAs to under-5 mortality and morbidity in the region. The review also highlighted the need to address diagnosis, prevention, surveillance and care to meet Sustainable Development Goals 3.2 and 3.8. The SSA sub-region faces unique challenges, including fragmentation of efforts that we hope to surmount through sSCAN via a multidisciplinary and multi-stakeholder approach.

## Introduction

Twenty-four percent of the world’s under-5 population live in sub-Saharan Africa (SSA) [1], yet this region accounts for 50% of all deaths in this age group [2]. Projections suggest that the United Nations’(UN) Sustainable Development Goals (SDG) aiming to reduce the under-5 mortality rate to less than 25 per 1000 live births by 2030 (SDG-3) may not be achieved by 51 countries, two thirds of which are in SSA [3,4]. Congenital anomalies (CAs) are a major contributor to the high child morbidity and mortality in SSA. They are defined as structural anomalies that occur during intra-uterine life and can be identified prenatally, at birth, during infancy and in some cases, adulthood [5]. CAs are a sub-set of the broader birth defects collective, which also includes functional disorders [6].

Of the estimated 8.5 million new cases of CAs that occurred globally in 2019, 30% were in SSA [7]. Ranked as the 5^th^ leading cause of death worldwide for children under-5 [4], CAs were responsible for an estimated 473,400 under-5 deaths in 2019 and represent approximately 9.4% of all under-5 deaths globally [7]. As progress continues to be made in addressing causes of infectious disease mortality in SSA, CAs will contribute greater proportion of child mortality. It is estimated that 90% of all severe CAs occur in low- and middle-income countries (LMIC) [5] and 38% of global deaths under-5 years due to CA are in SSA [7].

Despite the high burden of infant and under-5 mortality and morbidity related to CAs, these conditions appear to be a low priority on maternal and infant healthcare agendas in many SSA countries. From 2000 to 2015, the under-5 mortality rate due to CAs decreased by only 1% in SSA compared with 36% in North America and Europe [8]. There is no coordinated approach within the 49 countries of the sub-region to comprehensively address CAs and SSA remains the only region in the world without a CA surveillance network [9]. To address these gaps, SSA country representatives have established the sub-Saharan African Network for Congenital Anomaly Surveillance, Prevention and Care (sSCAN) [10]. To provide context for the activities of the Network, we conducted a scoping review to assess the current scope and depth of CA research within SSA.

Scoping reviews are a robust methodology to “systematically map the literature available on a topic” and to identify key concepts, theories and gaps in an area of research [11]. Such reviews explore the breadth and extent of available literature. They are often used to inform future research (as in this case) or as precursors to systematic reviews which are applied to more specific research questions [12–15].

The aim of this review is to determine the scope, objectives, and methodology of recent published research on CA in SSA, to categorize the focus of outputs (i.e., burden, surveillance, prevention, care) and identify potential stakeholders and researchers who may fall within sSCAN’s remit.

## Materials and methods

The approach used for this scoping review was based on the Arksey and O’Malley methodology [12] with modifications from the Joanna Briggs Institute [16] guided by the PRISMA-ScR via the Cochrane network [17].

### Eligibility criteria

**Fig 1:**
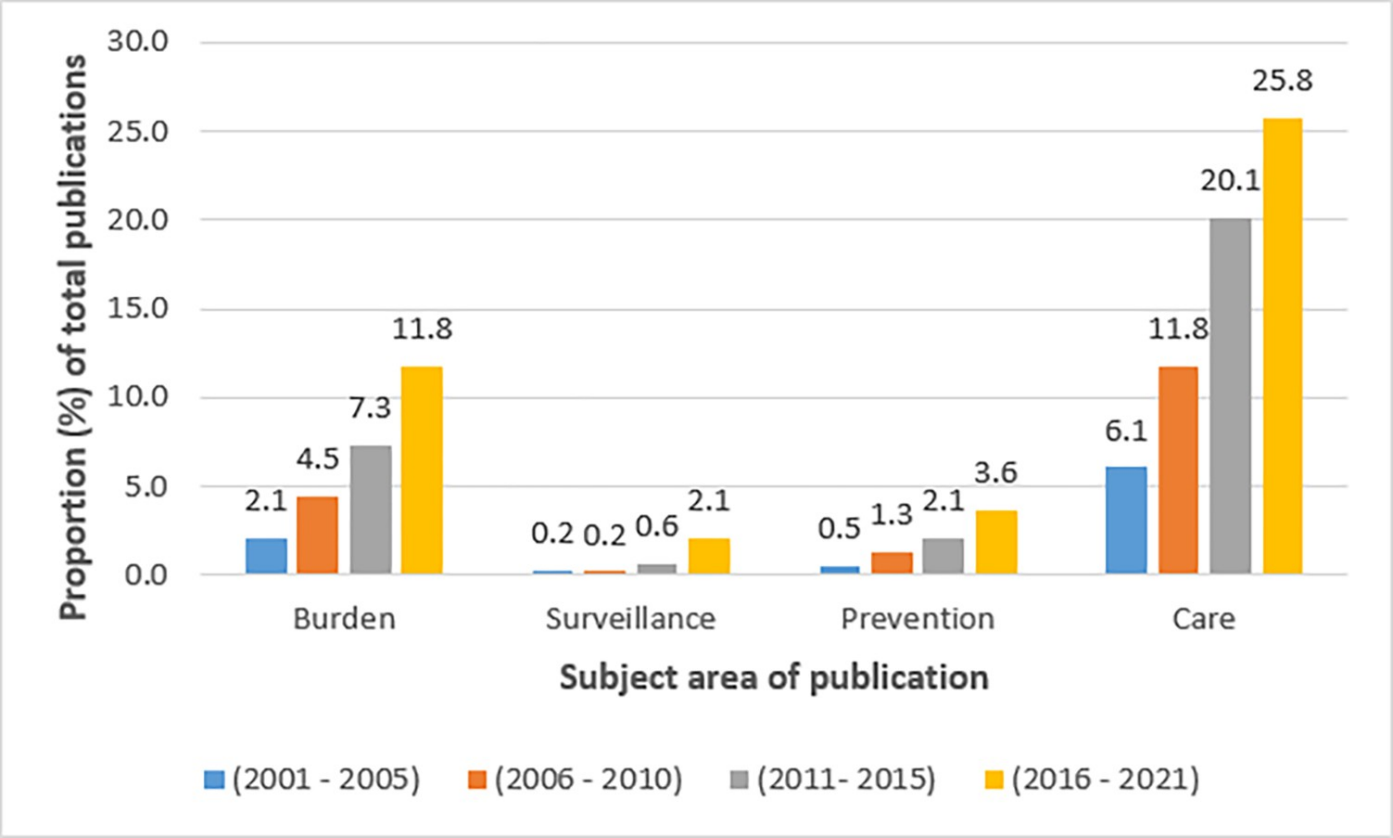
Trends in publication of papers related to CAs in SSA from 2001–2021, by subject area (subject areas were not mutually exclusive).

Original articles in English or French reporting on CAs in SSA as primary or secondary outcomes/objectives, focused on pregnancy (antenatal) care or involving infants or children <16 years, were included. An initial review to inform the study timeframe indicated that most articles were published in the last five years (Fig 1). Since sSCAN aims to consolidate current and future CA activities in SSA, the study period was limited to January 2016—June 2021. Systematic reviews were included to facilitate discussion. General literature reviews, editorials and letters lacking original data were excluded.

### Information sources and selection of papers

A detailed search was conducted in the Medline database, divided into four major sub-sections relevant to CA: 1) Burden, 2) Surveillance, 3) Prevention and 3) Care. The search included both MeSH and text terms. The following Boolean string searches were used for each major sub-heading:

1. **Burden** (Burden *OR* prevalence *OR* infant/child mortality *OR* years of life lost/YLL *OR* disability adjusted life years/DALY *OR* cost of care *OR* economics *OR* survival outcome *OR* Survival) ***AND*** (congenital anomalies *OR* congenital abnormalities *OR* birth defect)
2. **Surveillance** (Population Surveillance *OR* Public Health Surveillance *OR* Surveillance *OR* Epidemiological Monitoring) ***AND*** (congenital abnormalities *OR* congenital abnormalities *OR* birth defect)
3. **Prevention** (Prevention *OR* folic acid supplementation *OR* vaccination *OR* infection *OR* nutrition, *OR* diabetes *OR* alcohol *OR* medication *OR* drugs *OR* smoking *OR* risk factors) ***AND*** (congenital anomalies *OR* congenital abnormalities *OR* birth defect *OR* Neural tube defect/NTD *OR* Congenital Rubella Syndrome)
4. **Care** (care *OR* surgery *OR* stigma *OR* neglect *OR* culture *OR* beliefs *OR* Barriers to care *OR* Rehabilitation *OR* prenatal diagnosis) ***AND*** (congenital anomalies *OR* congenital abnormalities *OR* birth defect *OR* NTD *OR* hydrocephalus *OR* cleft lip/palate *OR* CHD)

Search results from each subsection were merged and imported in Reworks where duplicates were removed. It is important to note that our search terms were in English only, although all French papers that were retrieved were evaluated.

Articles were assigned to ten pairs of sSCAN co-authors (~53 articles/pair), who evaluated eligibility using title and abstract (primary screening). Full text was obtained for eligible articles only.

### Data abstraction

The co-author pairs undertook secondary screening of full-text articles and abstracted key data elements into a standardized Data Collection Form developed using Microsoft Excel and piloted and finalized by all authors (S1 Data). The co-authors categorised papers according to burden, surveillance, prevention, or care (not mutually exclusive), with sub-categories within these areas, and further classified papers by methodology. Disagreements within/between author pairs were resolved via discussion. To optimize inter-rater reliability, six articles in each pair were reviewed by all authors. See S2 Data for extracted data from papers reviewed.

The author team defined disease surveillance as “a continuous and systematic process of collecting, analyzing and interpreting health-related data in order to monitor disease progression and establish patterns in a defined population”. Papers originating from an ongoing surveillance programme were classified under surveillance, where two types were distinguished: (1) Independent surveillance programmes focusing exclusively on CAs, and (2) Public health surveillance programmes which involve routine data collected by the Ministry of Health from all care facilities and included CA amongst other conditions. Studies classified as independent or public health surveillance were expected to have originated from established, ongoing initiatives.

### Data analysis

Abstracted data were merged and cleaned in Microsoft Excel. Using STATA Statistical Software (Release 15. College Station, TX: StataCorp), categorical variables were described using frequency counts and proportions. Two authors conducted a thematic analysis in Microsoft Excel for descriptive, thematic presentation of the papers. Additional qualitative analysis of full-text articles was undertaken using Atlas ti Windows (Version 9) to code themes and determine the frequency of specific emerging key topics.

## Results

### Selection of articles

**Fig 2:**
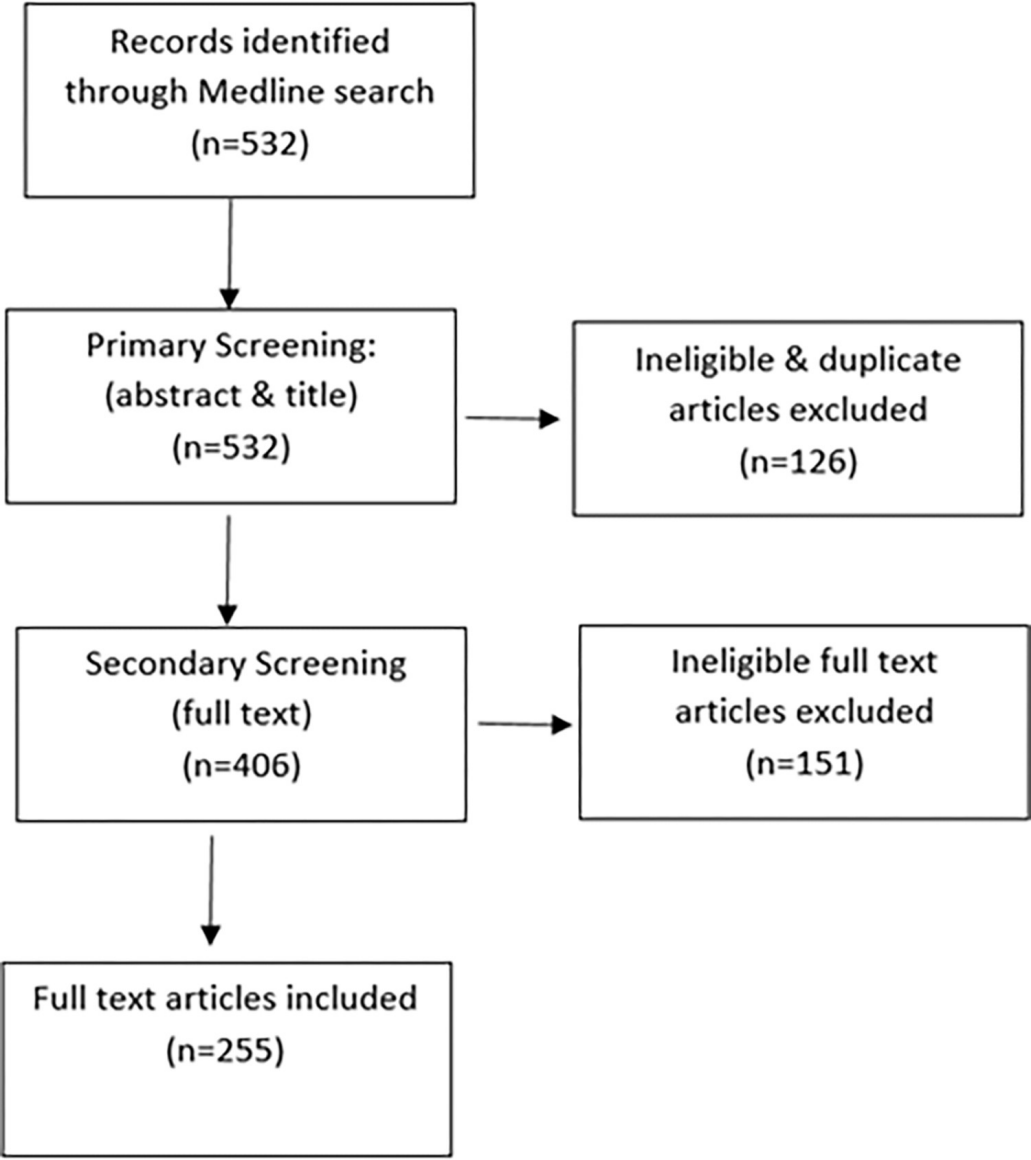
PRISMA study selection flow chart outlining screening process.

The initial search identified 532 articles published between January 2016 and June 2021. Of these, 255 [18–272] studies were included in the final review after several rounds of screening (Fig 2).

### Trend of publications

**Fig 3:**
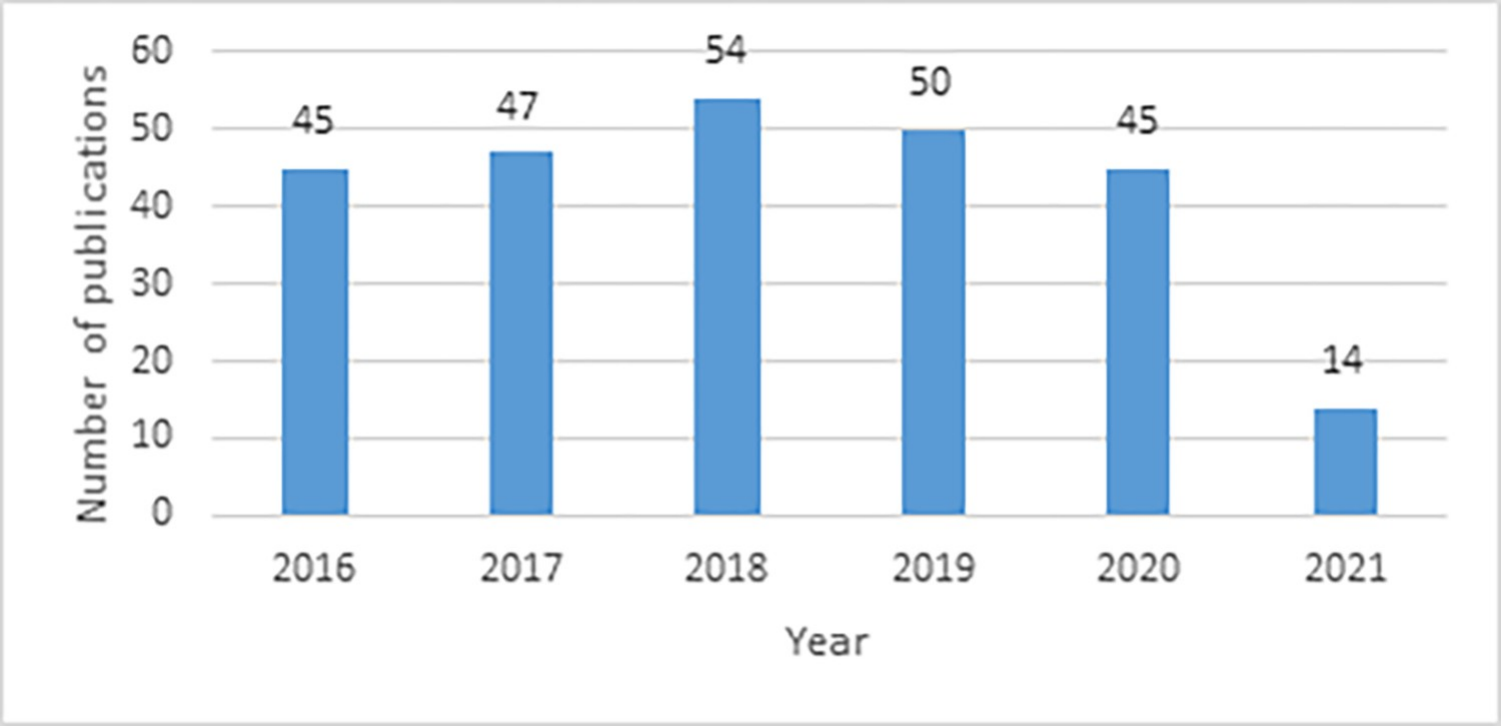
Number of publications per year (2016 –June 2021).

The number of publications remained relatively constant between 2016 and 2020 (Fig 3), with a drop in 2021 (2 per month rather than 4 per month on average), considering the study period ending in June 2021.

### Distribution of publications by country

**Fig 4:**
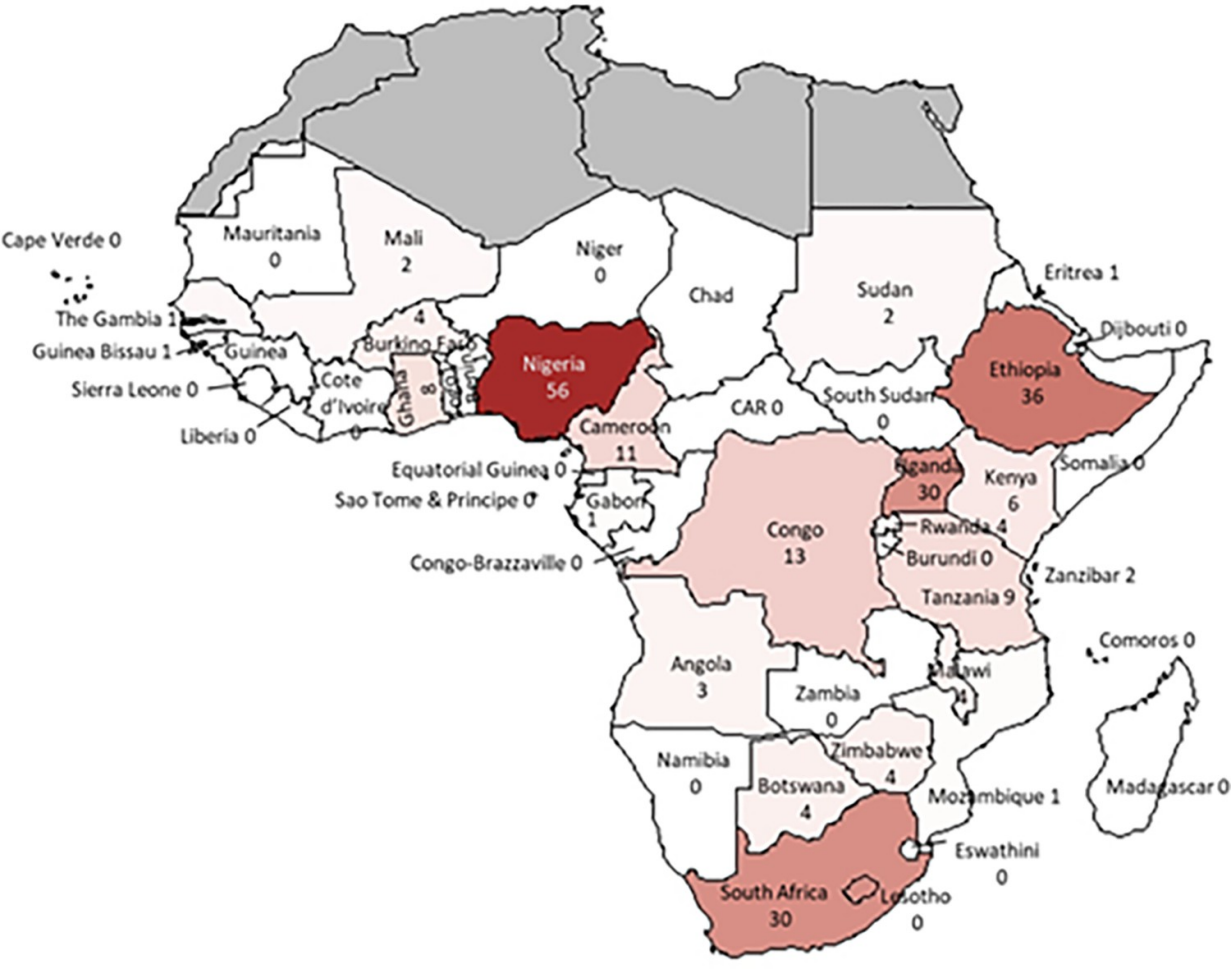
Number of articles reviewed by country (darker colour indicating higher number of articles). Source: Created using the R software (coordinates obtained using map_data and plotted using ggplot).

Final papers included originated from 26 of the 49 countries in SSA, with more papers from Eastern (38.6%) and Western (29.3%) Africa compared to Southern (17%) and Central (10.4%) Africa (Fig 4; S1 Table). Papers from Nigeria (22.0%), Ethiopia (14.1%), Uganda (11.7%) and South Africa (11.7%) accounted for almost 60% of the total. Several papers were global reviews including some SSA countries (4, 1.6%) or studies involving multiple countries within SSA (14, 5.5%).

### Types of CAs

**Fig 5:**
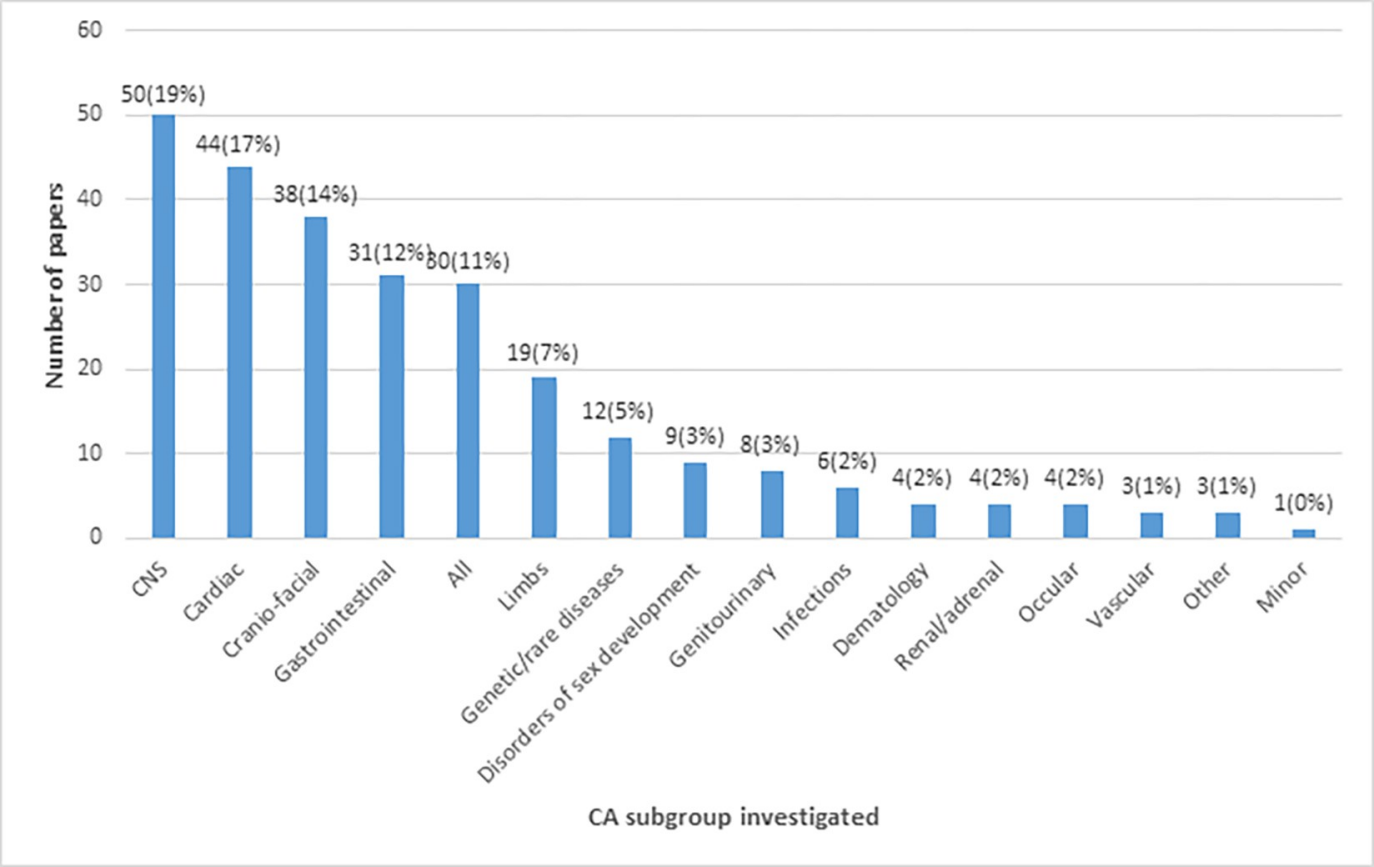
Number of publications per type of congenital anomaly.

Most of the papers reviewed had CA as their primary focus (85%) and investigated a single CA type (88%). Central Nervous System (CNS) (19%), cardiac (17%), craniofacial (15%) and gastro-intestinal (12%) anomalies were the four most investigated CAs (Fig 5). S2 Table shows breakdown of specific CA types investigated.

### Focus areas and objectives

**Fig 6:**
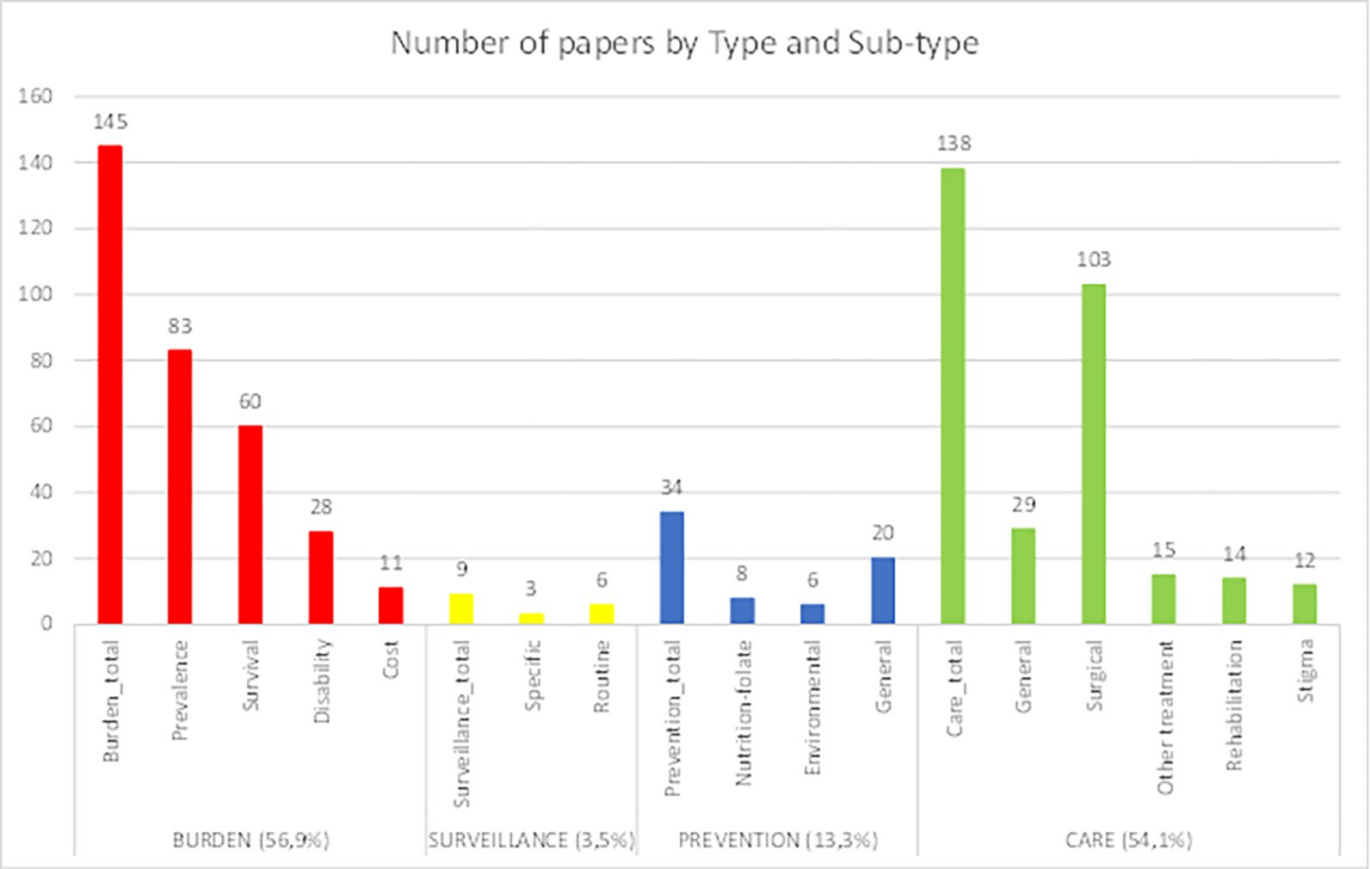
Distribution of papers across the four key categories.

Fig 6 shows the distribution of articles across the four major subject areas (burden, surveillance, prevention, and care), with a further breakdown into sub-areas. Single case studies were excluded from the thematic analysis.

**a. Burden**

A total of 145 (56.9%) papers addressed issues related to burden, making it the most published topic.

*i. Prevalence*

Studies of prevalence (n = 83) provided data regarding the prevalence at birth or during childhood (e.g., among school children) of specific CAs (n = 60) or a range of CAs (n = 23). Most were hospital-based studies, either from a single (n = 37) or multiple (n = 27) hospitals. A few were population-based studies (e.g., [60,61,94]), and surveys of school children (e.g., [33,63,184]). Reporting on prevalence was often a secondary objective of single hospital studies addressing surgery and care, or of cohort or case-control studies of risk factors and prevention. Prevalence data were accompanied by additional descriptive epidemiology, such as sex ratio, maternal age distribution or other sociodemographic variables, and the clinical characteristics of cases.

*ii. Survival*

Survival papers mainly looked at survival of babies with CA admitted for surgical care (e.g., [19,28,54,66,76,89]). The period of survival was mainly during the first year of life, or in the days/months following surgery, with no long-term survival studies. In some studies, mortality of babies with CAs was included as part of an investigation into all causes of neonatal death (e.g., [29,150,264]). In others, mortality was reported as an outcome following implementation of a new care protocol (e.g., [54]); or as rates of termination of pregnancy for fetal anomaly following prenatal diagnosis (e.g., [84,87]). Most studies (88.3%) investigated survival for a single CA, of which neural tube defects (NTD; hydrocephalus/spina bifida (25.0%) and congenital heart disease (CHD; 18.3%) were the most common, followed by gastrointestinal anomalies such as atresias (15%) and gastroschisis (5%).

*iii. Disability*

Papers focusing on disability generally investigated how living with untreated CA affected physical/functional capacity (e.g., [63,103,109,134]), with some reporting on associated comorbidities (e.g., [64,75]). One paper [134] evaluated self-reported quality of life of children living with spina bifida compared to unaffected children. A few papers investigated the impact on the quality of life for mothers (e.g., [30,195,256]) and caregivers of affected children (e.g., [25,31]). One paper [214] derived disability weights, used to calculate Years Lived with Disability (YLD). Orofacial clefts (OFC) (21.4%) were the most CA investigated in relation to disability, with clubfoot (7.1%) and intestinal atresia (7.1%) also noted.

*iv. Cost of Care*

For the 11 papers identified for this category, the focus was upon cost of care in relation to financial challenges influencing access to care and poor outcomes (e.g., [25,31,89,236]), alongside social challenges [31,175]. Papers on cost of care primarily focused on CHD (45.5%) and OFC (27.3%), relating to the financial burden of individuals accessing care (and the impact on care seeking) rather than the cost to the health system. A further 105 papers mentioned the financial burden of CA facing families but did not collect or analyse data on this issue.

**b. Surveillance**

Papers that reported on long-term ongoing collection and analysis of CA data were categorized as surveillance. These reported on prevalence (included also in Burden), or on risk factors (also included in Prevention). Surveillance data originated from the routine collection of data by the public health system across many diseases (n = 6 [60,77,78,94,129,249]), or from specific independent initiatives (n = 3 [86,99,100]). Eight of the nine surveillance papers addressed a single type of CA (spina bifida (n = 4), congenital rubella syndrome (n = 3) and microcephaly (n = 1). A further 38 (15%), mentioned surveillance, mainly in relation to the need for more comprehensive surveillance of CA.

**c. Prevention**

Thirty-four (13.3%) papers addressed issues related to prevention, either known risk factors such as folic acid, or other potential causes of CA.

*i. Folic Acid*

Eight (23.5%) of the 34 papers on prevention focused on folic acid, most commonly reporting on the use of folic acid by mothers in the context of NTD and/or hydrocephalus (e.g., [22,105,123,124,132,164]). Four papers focused on barriers to [34], awareness of the benefit of [49], and estimation of potential reduction in the rate of NTD and hydrocephalus due to folic acid use [94]; and modelling of potential prevention of under-5 mortality [94]. A further 47 (18.4%) papers mentioned folic acid but did not collect specific data.

*ii. Maternal infections including Rubella*

Six papers focused solely on rubella or rubella vaccination [60,129,145,176,232,249], addressing rubella infection prevalence among women, immunity in adolescents, screening, and the baseline prevalence of congenital rubella syndrome prior to the introduction of immunization (60). A further 17 papers mentioned rubella or rubella vaccination but did not collect specific data [26,34,55,64,70,73,78,83,88,123,153,164,165,192,238,242,244]. One paper investigated the emergence and circulation of Zika virus in SSA [78].

*iii. Environmental Exposures*

The two main categories of risk factors investigated were medication safety in pregnancy (n = 8, [70,96,99,100,123,159,164,207]), and environmental pollution (n = 6). Most medication safety studies evaluated a series of medications (including herbal) as part of a general CA risk factor assessment [70,96,123,164], while a few evaluated the safety of a single medication type/class (antiretrovirals [99,100] and antimalarials 207) or general medication use in pregnancy [159]. There were no papers on common medicines used for non-infectious diseases, or on the impact of vaccination during pregnancy. Herbal remedies and traditional medicine featured prominently in 34 papers, including those which did not collect data.

Papers on environmental pollution focused particularly on exposure to extraction metals such as copper, cobalt, mercury and arsenic [67,70,71,95]. A further 16 papers mentioned environmental pollution, but did not collect specific data [26,34,44,50,67,70,71,88,95,96,105,132,160,164,183,216,222,227,232,237].

A cluster of 13 papers specified radiation exposure during pregnancy as a concern [19,33,34,62,70,76,105,107,132,148,188,202,203].

**d. Care**

Care was the second most common area investigated after burden, and the focus of 138 (54.1%) papers, mainly aimed at describing and improving treatment and care outcomes.

*i. Surgical care*

There were 103 papers related to surgical care, of which 36% focused on surgery outcomes. The main surgical areas were cardiac (23.4%), CNS (for NTD and hydrocephalus; 20.8%), gastrointestinal (predominantly anorectal malformations; 18.8%), OFC (14.9%) and orthopedics (predominately congenital talipes; 11%). Papers focused on different issues, including challenges faced by patients in accessing surgical care e.g. [25,35,37] (including due to COVID-19, [57]); and performing surgery for complex anomalies [37,38]; the quality of peri- and/or post-operative surgical care (e.g., [32,33]); outcome/survival after surgery (e.g., [19,89,120]); and developing cost-effective, local surgical management tools (e.g. management silos for gastroschisis [58]). Notably, late or missed CA diagnosis either due to late presentation, lack of access to care, or shortage of diagnostic expertise was addressed in several papers (e.g., [28,81,91,110,114]). For example, one paper investigating access to care by caregivers of children with congenital heart defect (CHD) concluded, “Delayed access to care was largely due to the lack of early CHD recognition and financial hardships related to the inefficient and disorganized health care system” [25]. Papers also reported case series, including patient characteristics, and clinical characteristics and complications, to aid service planning and improvement.

*ii. Rehabilitative care*

Papers on rehabilitation constituted 5.5% of all papers reviewed and addressed issues such as evaluation of training of rehabilitation care professionals and the quality of care provided (e.g., [20,46]), effectiveness of specific rehabilitation techniques (e.g. [91,109]), characteristics of children with untreated CA presenting for rehabilitative care (75), and opinion of caregivers regarding cosmetic care (e.g. plastic surgery) (e.g. [102]). OFC (42.9%) and clubfoot (35.7%) were the most common anomalies investigated in relation to rehabilitative care. Speech therapy and plastic surgery were common topics included for children with OFC.

*iii. Stigma and related cultural issues affecting care*

Papers on stigma and related cultural issues (n = 12) were often based on surveys administered to care givers (mostly mothers) of children with CAs and addressed issues such as the influence of stigma on the quality of care (e.g., [30,34]), social/cultural barriers to uptake of preventive measures [31], prenatal screening [116] and treatment [31], genital reconstruction in disorders of sex development [120,128], plastic surgery [102], community rejection and abandonment by spouse [31,175], as well as health system responsiveness [175]. Some papers documented family and community beliefs about the causes of CA, e.g., witchcraft, contraception, and the guilt of the mother [31,116,241]. Beliefs and perceptions about the efficacy of medical care, preference for traditional medicine [241], and parental/community believes on whether treatment should be sought [31,241] were also included.

*iv. Other themes relating to Care*

Eight of the care papers [20,46,57,72,114,154,180,258] were system-focused rather than addressing specific care, and these included training, professional development and service delivery, and multidisciplinary teams. These described how training and service organization initiatives have improved care.

Seven papers reported on the experiences of healthcare of parents and caregivers, and their healthcare-seeking behaviours [25,31,44,102,166,179,241]. These studies interviewed parents and caregivers to find out about their navigation of the healthcare system, documenting difficulties in obtaining a diagnosis, and accessing specialist care.

Fourteen papers reported on dysmorphology or genetics [42,56,59,103,120,128,133,170,210,212,224,246,271]. These studies undertook detailed examinations of babies with CA to understand the range of dysmorphologies, and the associations between different anomalies. One paper focused on ethnic differences in minor anomalies [42]. Several papers reported the genetic distribution of disorders of sex development [59,120,128,224].

Seven papers investigated the results of prenatal diagnostic services [65,84,87,116,161,188]. These addressed the problem of limited information about prenatal screening of CA via ultrasound, including how often it is conducted, role of counseling, rate of parents’ acceptance of the screening and termination when a diagnosis is made, as well as its overall value supporting early diagnosis and care.

### Methodologies

Six aspects of study methodology were examined: study type, population coverage, data source, type of birth, age at CA diagnosis, and expert confirmation of CA diagnosis (Table 1).

**Table 1:** Proportion of studies across different methodologies.

Method element	n (%)
**Type of study**	
Case study/case series ^a^	67 (26.6)
Cross-sectional survey	45 (17.6)
Retrospective record review	44 (17.3)
Cohort study	44 (17.2)
Case-control study	16 (6.3)
Descriptive Epidemiology	11 (4.3)
Diagnostic study	8 (3.1)
Systematic review	7 (2.7)
Qualitative study	6 (2.3)
Economic/costing	2 (0.8)
Modelling	2 (0.8)
Intervention	2 (0.8)
Other^b^	1 (0.4)
**Population coverage**	
Single hospital	154 (60.4)
Multiple hospital	53 (20.8)
Population-based	23 (9.0)
Not given	25(9.8)
**Data source**	
Clinical records	143 (56.1)
Interviews with care-givers	90 (35.3)
Published literature^c^	9 (3.5)
Surveillance data	5 (2.0)
Laboratory-based (biological samples)	3 (1.2)
Smile Train database	2 (0.8)
Recommendations	1 (0.4)
Other^b^	1 (0.4)
**Type of birth**	
Live birth	195 (76.5)
Still birth	19 (7.5)
Termination of Pregnancy	6 (2.4)
Not given	50 (19.6)
**Age of diagnosis**	
Any	45 (17.6)
At birth	51 (20.0)
Child	68 (26.7)
Prenatal	9 (3.5)
Not given	70 (27.5)
**Expert confirmation of diagnosis**	
Yes	150 (58.8)
No	12 (4.7)
Not given	79(31.0)

^a^ Includes 25 single case studies.

^b^ *Development of a low-cost material for the treatment of gastroschisis*.

^c^ *Systematic review*.

Case studies/case series (26.6%) were the most common study type with a considerable number of cross-sectional surveys (17.6%), retrospective record reviews (17.3%), and cohort studies (17.2%). For population coverage, most studies (60.4%) were conducted in a single hospital, with few population-based studies (9%). Data were mainly sourced from retrospective review of clinical records (56.1%) or via interviews with caregivers (34.9%). Very few papers included stillbirths (7.5%), TOPFA (2.4%) or prenatally diagnosed CAs (3.5%). For most papers (58.8%) the CA diagnosis was confirmed by an expert.

## Discussion

### Trends in CA-related publications in SSA

This scoping review revealed an increase in CA-related publications in SSA within the last decade. This may reflect increased awareness among researchers and stakeholders during this period, stemming from CA-related events such as: 2010 World Health Assembly (WHA) Birth Defects Resolution 63.17 that called for the strengthening of birth defect surveillance systems globally and nationally [273]; and the series of birth defect surveillance training sessions in SSA organized by the International Clearinghouse for Birth Defects Surveillance and Research (ICBDSR) in 2014 and 2015 [274]. This publication trend mirrors the proportional increase of CA as a cause of childhood mortality and morbidity as infectious causes decline. For example, from 2000 to 2017, the number of under-5 deaths in Africa due to CAs increased by 31,000 (19%), in contrast to HIV/AIDS and measles for which under-5 deaths decreased by 144,000 (-68%) and 277,000 (86%), respectively [8].

Most studies addressing CAs included in the review were undertaken in a handful of countries, with 66% of countries in the region publishing no research on these conditions. This highlights the extent to which CA-related research is neglected in SSA despite the high disease burden. Most studies were confined to specific countries/institutions with minimal cross-border collaboration to support and strengthen surveillance and care. Disseminating research on CAs and their relative contribution to childhood mortality and morbidity is vital to provide an evidence base for investment in initiatives aimed at primary, secondary and tertiary prevention. This review highlights the importance of initiatives such as sSCAN in building awareness around the burden of CAs in the region, creating a network of support and collaboration for researchers and clinicians, and advocating for better access to care.

### Delay in diagnosis and the impact on care outcomes

As expected, the majority of CAs featured in this review are visually obvious, external conditions, including NTD and other CNS conditions, cranio-facial and gastrointestinal anomalies. While CHD also featured, the burden of these internal and other less obvious anomalies and functional congenital disorders (single gene disorders and rare diseases) requiring additional diagnostic tools continue to be under-diagnosed and under-reported due to misdiagnosis and premature mortality incorrectly reported. This prevents an accurate assessment of the contribution of these conditions to the disease burden. Literature is lacking on survivorship and the proportion of those receiving an accurate diagnosis and relevant treatment and developing this baseline essential.

Lack of screening is a major contributor to the high rate of missed and delayed CA diagnosis in SSA. While conducting a surface examination of all newborns is standard practice, it requires appropriate skills and capacity. In an attempt to address this, the WHO/CDC/ICBDSR has recently launched a birth defect surveillance toolkit with checklists to improve efficiency and data quality [275]. Recent reports indicating many SSA countries are gradually implementing pulse oximetry for postnatal screening of CHD [276] is encouraging, although poor performance concerns related to use of this technology in darker-skin babies are emerging [277,278]. High resolution, prenatal ultrasound screening for CAs remains limited in LMIC due to the expensive equipment and skilled personnel required. Recent advances in technology offer low-cost, compact mobile ultrasound devices, dramatically increasing the availability of ultrasound services in LMICs [279,280]. The expertise required to perform a fetal anomaly ultrasound scan still presents a major barrier. Emerging artificial intelligence solutions, automatically detecting CAs and capturing accurate images may allow less-skilled personnel to perform the procedure in the future [281].

### Training, service development and surgical care

Given the rising burden of CAs and lack of expertise in SSA, there is an urgent need to increase clinical capacity to treat correctable CAs. While the number of paediatric surgeons in SSA is increasing, numbers of other important personnel such as specialist paediatric surgical nurses, intensive care personnel, paediatric anaesthesiologists, clinical geneticists, radiologists, and neonatologists remain low.

Historically, surgical camps or global outreach teams have filled this gap, but these are less sustainable than upskilling local providers, and often reach only a few of those requiring care [282,283]. While some initiatives transfer skills and develop local expertise, many make little local impact. To succeed, such initiatives must be locally driven by individual champions in SSA with continued capacity-building of local teams following the intervention. Some collaborative efforts with HIC-based organizations have seen significant success in the treatment CAs in SSA. These are often single disease programmes, such as Smile Train (www.smiletrain.org)) which focuses exclusively on OFC.

### Awareness and access to care

Poor health literacy and education across SSA is a significant barrier to navigating the health system, especially for those impacted by CA. This is compounded by the lack of well-coordinated care for babies diagnosed with CA. Knowing how, where and when to access appropriate care, especially in rural areas with vast geographical distances may be overwhelming. Specialist treatment centers are mainly located in urban centres, significant distances away from birthing centres. Long-distance travel on poor roads with inadequate public transport infrastructure pose a risk to vulnerable infants, in addition to the cost implications for the family. Most families in SSA live below the poverty line and cannot cover out-of-pocket healthcare costs, combined with additional living costs, childcare for siblings and lost earnings. Seeking care for the child affected by a CA may require overcoming insurmountable barriers.

State-funded insurance schemes and government subsidies could dramatically improve the care of affected children combined with telemedicine to facilitate remote care, limiting costs and travel. The most pressing need is for CAs to be integrated into mainstream primary health care systems in SSA, with a well-coordinated system to promote prevention, screening, diagnostics and care. This could be championed by initiatives such as sSCAN in collaboration with governments, local organizations, patient advocacy and support groups and other external stakeholders.

### Surveillance and prevalence data

Studies assessing prevalence in this review reflected the challenges faced in establishing the baseline birth prevalence of CAs in the sub-region. Most studies involved hospital-based data, predominantly in a single hospital, with only three studies [60,61,94] assessing population-based prevalence. High rates of home-based deliveries, limited diagnostic capacity and clinical expertise are only a few of the barriers researchers face in determining the true burden of CAs in SSA. The few existing surveillance projects have been driven by the need to assess medicine safety during pregnancy such as antiretrovirals and antimalarials. Surveillance systems in Botswana, South Africa, Malawi, and Uganda have focused on assessing the safety of antiretrovirals in pregnancy and birth outcomes, including CAs. As the pipeline of novel medicines, vaccines and other health technologies expands, such projects are likely to increase in the region, providing an opportunity to build capacity around CA surveillance and generate much needed empiric data on CAs [284].

### Prevention

Primary prevention (i.e. preventing CA-affected pregnancies) is the ideal approach to reducing the CA burden of disease [285]. This requires optimized prenatal and perinatal nutrition (e.g., folate fortification to decrease NTD risk), reduced exposure to known teratogens (e.g. infections, alcohol, prescribed and recreational drugs), and research to establish the teratogenicity of environmental exposures where evidence is lacking or limited. A broad, integrated primary prevention strategy for CAs in SSA should be envisaged. This review revealed limited research activity in this area.

In addition to reducing risk factors (e.g. maternal diabetes, infections, toxic exposures) improving maternal folate status by food fortification is a highly effective primary prevention strategy. This has been proven to prevent a substantial fraction of severe NTDs, especially spina bifida and anencephaly, as well as addressing anaemia [286]. Six of the 18 countries identified for their potential to maximize reduction in anemia and NTD through large-scale fortification are in SSA. Encouragingly, this scoping review has identified several publications addressing aspects of folic acid-related prevention. However, much more can be done to help translate the promise of folic acid into meaningful reduction in NTDs.

Secondary prevention strategies reduce the number of CA affected births (including pre-natal diagnosis, genetic counselling and option of TOPFA while tertiary prevention strategies involve the early diagnosis of CA (even prenatal) with prompt treatments to prevent complications and mitigate disability [287].

### Risk factors

The limited scientific evidence-base related to CA associated with medication use in early pregnancy is a global issue, but in SSA there are additional concerns relating to the wide-spread use of essential medicines for the prevention and treatment of infectious diseases such as malaria and HIV [288,289], and the high prevalence of herbal medication use [290]. Inadequate research on medication safety in pregnancy may delay access by pregnant women to treatments with an optimal benefit-risk profile for both mother and baby. There is also a need to monitor and respond to the use of medicines with a known teratogenic profile.

Maternal infections are a well-established cause of CAs. However, other than rubella, the only other infectious disease related to CAs included in this review was the Zika virus. First recorded in Uganda, Zika has received global attention following an epidemic in Latin America starting in 2015, causing severe microcephaly [291]. The extent of microcephaly risk relating to Zika in Africa, where both the specific virus and mosquito differ from those in Latin America, is unknown due to the lack of functioning surveillance systems in the sub-region. Other diseases requiring more attention include congenital syphilis [292], toxoplasmosis [293,294], and cytomegalovirus [295,296], due to their higher prevalence in SSA. Compounding effects of multiple infections (co-morbidities) affecting the fetus, and interaction with malnutrition and other exposures also require consideration.

Environmental air, water and soil pollution is an ongoing concern with particularly high levels reported in African countries of heavy metals and pesticides [297]. Five studies in this review researched environmental pollutants in relation to CA risk, three of which were undertaken due to widespread contamination from mining operations [67,71,95].

No studies on dietary factors (other than folic acid) were included in this review, but several, very specific practices such as “chewing of khat” [70] were identified. While Fetal alcohol spectrum disorder (FASD) is a significant public health problem in many communities, including in SSA [298], this review did not include search criteria to identify studies addressing alcohol or smoking during pregnancy. Due to the difficulty in diagnosing FASD at birth, such a review would not be comprehensive. With the increasing prevalence of obesity and diabetes in African countries [299], periconceptional management of diabetes to prevent CHD and other CA is becoming more important. No papers in the review addressed this issue.

Although this review did not investigate the relationship between socioeconomic status and CAs, it was noted that extreme poverty is particularly prevalent in rural areas of SSA, where it is most difficult to obtain reliable data on CAs caused by a combination of nutritional deficiencies, infections, and environmental exposures. It can therefore be concluded that poverty both causes CA and is associated with poor access to treatment and poor outcomes.

### Influence of culture

Few papers in this review addressed issues related to stigma and culture. SSA is one the most culturally diverse region of the world [300], with deeply rooted cultural and social underpinnings that influence health-seeking behavior. Across the region, the process of pregnancy and birth is regarded as “secret” and associated with varied cultural and spiritual dimensions. Notably, the birth of a baby with CA often carries stigma and varied cultural/social interpretations as to the cause and approaches to treatment. Strategies to address CA in SSA must therefore include robust attempts to fully understand associated cultural and social challenges and to develop mitigation frameworks that include holistic approaches involving the entire community. A starting point could be to learn from the challenges and successes of similar stigma addressing related conditions such as HIV/AIDS [301]. Particular attention must also be given to the cultural and social factors that influence the use of traditional medicines during pregnancy and for the management of CAs. This has become even more urgent with the current WHO plan to incorporate traditional medicine into mainstream healthcare systems in SSA [302].

### Health information systems

Comprehensive, formalized and centralized electronic health record (EHR) systems are scarce in SSA, seriously impacting the availability of empiric surveillance data for CAs. This is highlighted by the limited literature reporting on surveillance systems and the dominance of hospital record reviews and interviews as data sources. The lack of EHR systems makes wide-scale linkage of pregnancy exposures and neonatal outcomes extremely challenging. There is a need to build awareness around the importance of accurate, quality data as an evidence-base to better inform resource allocation and clinical decisions, and to motivate for investment in EHR systems in the region.

The high rates of out-of-facility deliveries in some countries, fragmented obstetric services and resource constraints with respect to expertise, funding, and information and communication technology (ICT) challenge the implementation and maintenance of EHRs [303,304]. In the last 20 years there has been remarkable improvement in the ICT infrastructure across SSA, and the proliferation of mobile devices [304]. Recently, the African Union expanded its Digital Transformation Strategy for Africa for a further 10 years. These developments present an exciting opportunity to improve upon EHR data capture. Within such a resource-constrained environment even minimal expansion of system coverage could be implemented for targeted disease areas, as has been the case with HIV/AIDS [305]. Uptake of innovative tools such as the new Global Birth Defects app specifically designed to facilitate diagnosis of CAs for surveillance programs operating in low resource settings such as SSA [306], should be maximized.

### Strengths and limitations

This is the first scoping review investigating recent literature on CA in SSA. It was conducted to inform the activities of the newly established sSCAN and designed to maximise network involvement evaluating and interpreting the literature. This approach may have led to inconsistencies in the categorization of papers between data abstractor groups. While this was mitigated as far as possible via the use of a standardized data extraction form accompanied by specific instructions and training, misclassification remains a possibility.

The results of the literature search were dependent upon the inclusion of CA and associated MESH terms as key words in the literature. Some papers on risk factors may have been missed if they were not specifically related to CA. For example, whereas maternal infections are risk factors for CA, but papers on maternal infections would only be included in this review if they related this risk factor to CA.

To facilitate reporting, papers were categorized into four main groups identified by author consensus. This categorization may be perceived as being arbitrary. For example, papers on stigma were allocated to a sub-category under Care but may just as easily be considered to relate to the sub-category of disability under burden. Similarly, cost of care was categorized under burden, but many papers related to financial costs to the patient/family and were a major element in papers regarding Care. This was mitigated by describing details on the objectives of papers and concerns of the authors in each category. The categories were also not mutually exclusive, and so papers could be allocated to multiple categories.

The timeframe of this review was limited to a five-year period to represent the “current” situation regarding research activity relating to CA in SSA, and the situation following the 2010 WHA Resolution 63.1. It was not our purpose to review all literature regarding CA in SSA. Additionally, the focus on obvious CA, a sub-set of congenital disorders, excluded less obvious structural anomalies as well as functional conditions, including most single gene disorders [6].

## Conclusion

There is increasing awareness and recognition among researchers in SSA of the growing contribution of CAs to under-5 mortality and morbidity in SSA together with the need to address diagnosis, prevention, surveillance and care of these conditions to meet SDG3.2 and 3.8 2030 targets [3]. SSA has specific challenges in relation to CAs and needs to formulate an approach to address these. The newly formed sSCAN can champion this course through a comprehensive, multidisciplinary and multi-stakeholder approach that involves national governments, local institutions, patient organizations, and relevant international organizations such as WHO, to ensure that opportunities for primary prevention are fully implemented and those impacted by CA receive optimal care and are not left behind.

## Supporting information

S1 Table Regional distribution of studies across sub-Saharan Africa. (DOCX)

S2 Table Congenital Disorders (not mutually exclusive). (DOCX)

S1 Data Data extraction template. (XLSM)

S2 Data Data extracted from papers reviewed. (XLSX)
