# Supplementary material for: The burden, prevention and care of infants and children with congenital anomalies in sub-Saharan Africa: A scoping review
Source: PLOS Glob Public Health. 2023 Jun 28;3(6):e0001850. doi: 10.1371/journal.pgph.0001850 (PMC10306220; doi:10.1371/journal.pgph.0001850)
Supplement: S1 Table — (DOCX) [file pgph.0001850.s001.docx]

| **Region n (%)** | **Country** | **n (%)** |
| --- | --- | --- |
| **Global** | Global | 4 (1.5%) |
| **Sub-Saharan Africa** | Regional | 14 (5.4%) |
| **Southern 44 (17.0%)** | Angola | 3 (1.2%) |
|  | Botswana | 4 (1.5%) |
|  | Lesotho | 0 |
|  | Mozambique | 1 (0.4%) |
|  | Namibia | 0 |
|  | South Africa | 30 (11.5%) |
|  | Swaziland | 0 |
|  | Zambia | 0 |
|  | Zimbabwe | 4 (1.5%) |
| **Western 75 (29.3%)** | Benin | 1 (0.4%) |
|  | Burkina Faso | 4 (1.5%) |
|  | Cape Verde | 0 |
|  | Cote D’Ivoire | 0 |
|  | Ghana | 8 (3.1%) |
|  | The Gambia | 1 (0.4%) |
|  | Guinea Bissau | 1 (0.4%) |
|  | Liberia | 0 |
|  | Mali | 2 (0.8%) |
|  | Mauritania | 0 |
|  | Niger | 0 |
|  | Nigeria | 56 (21.5%) |
|  | Senegal | 2 (2.3%) |
|  | Sierra Leone | 0 |
|  | Togo | 1 (0.4%) |
| **Eastern 100 (38.6%)** | Burundi (as part of regional only) | 0 |
|  | Comoros | 0 |
|  | Djibouti | 0 |
|  | Ethiopia | 36 (13.9%) |
|  | Eritrea | 1 (0.4%) |
|  | Kenya | 6 (2.3%) |
|  | Madagascar | 0 |
|  | Malawi | 4 (1.5%) |
|  | Mauritius | 0 |
|  | Reunion | 0 |
|  | Rwanda | 4 (1.5%) |
|  | Seychelles | 0 |
|  | Somalia | 0 |
|  | Somaliland | 0 |
|  | Sudan | 2 (2.3%) |
|  | Tanzania | 9 (3.56%) |
|  | Uganda | 30 (11.5%) |
|  | Zanzibar | 2 (0.8%) |
| **Central 27 (10.4%)** | Cameroon | 11 (4.2%) |
|  | Central African Republic | 0 |
|  | Congo-Brazzaville | 0 |
|  | Democratic Republic of Congo | 13 (5.4%) |
|  | Equatorial Guinea | 0 |
|  | Gabon | 1 (0.4%) |
|  | Sao Tome & Principe | 0 |
