# Supplementary material for: The burden, prevention and care of infants and children with congenital anomalies in sub-Saharan Africa: A scoping review
Source: PLOS Glob Public Health. 2023 Jun 28;3(6):e0001850. doi: 10.1371/journal.pgph.0001850 (PMC10306220; doi:10.1371/journal.pgph.0001850)
Supplement: S2 Table — (DOCX) [file pgph.0001850.s002.docx]

| Category n (%) | Congenital Anomalies |
| --- | --- |
| All congenital anomalies 30 (11.5%) |  |
| Minor congenital anomalies 1 (0.4%) |  |
| CNS 50 (19.2%) | Spina bifida |
|  | Anencephaly |
|  | Encephalocele |
|  | Hydrocephalus |
|  | Microcephalus |
|  | Chiari Malformation |
|  | Joubert’s Syndrome * |
|  | Cerebral Palsy |
| Cranio-facial 38 (14.6%) | Cleft lip/palate |
|  | Dental |
| Gastro-intestinal 31 (11.9%) | Oesophageal atresia |
|  | Intestinal atresia |
|  | Gastroschisis |
|  | Congenital bowel obstruction/Hirschprung’s Disease |
|  | Omphalomesenteric fistula |
|  | Duodenal diaphragm |
|  | Biliary atresia |
|  | Prune belly |
|  | Anorectal malformation |
|  | Cantrell Syndrome* |
| Genito-urinary 8 (3.1%) | Hypospadias |
|  | Cryptorchidism |
|  | Vaginal canal* |
|  | Recto-vestibular fistula* |
|  | Bicornate uterus* |
| Limbs 19 (7.3%) | Congenital talipes |
|  | Congenital hand anomalies |
|  | Congenital foot and ankle anomalies |
|  | Pes planus |
|  | Limb duplication* |
| Genetic diseases 12 (5.0%) | Trisomy 21 |
|  | Fragile X syndrome |
|  | Turner Syndrome* |
|  | Tuberos sclerosis |
|  | Sickle cell disease |
|  | Phacomatosis pigmentovascularis* |
|  | Holt-Oram syndrome* |
| Dermatology 4 (1.5%) | Congenital ichthyosis |
|  | Collodion baby* |
| Disorders of sex development 9 (3.5%) | Ambiguous genitalia |
|  | Congenital adrenal hypoplasia |
|  | Hypogonadism |
| Renal/adrenal 4 (1.5%) | Polycystic kidney disease |
|  | Congenital adrenal hypoplasia |
| Vascular 3 (1.2%) | Vein of Galen malformation* |
|  | Lymphatic malformation |
|  | Vascular ring |
| Cardiac 44 (17.4%) | Congenital heart disease |
|  | Bicuspid aortic valve |
|  | Thoracic ectopic cordis* |
| Infections 6 (2.3%) | Congenital rubella |
|  | Zika virus |
| Ocular 4 (1.5%) | Blindness |
|  | Ocular anomalies |
|  | Uveal coloboma |
|  | Pre-papillary vascular loops |
| Other 3 (1.2%) | Pulmonary airways malformations* |
|  | Sternal cleft* |

* Single case reports only
